# Terlipressin for the treatment of septic shock in adults: a systematic review and meta-analysis

**DOI:** 10.1186/s12871-020-00965-4

**Published:** 2020-03-05

**Authors:** Lili Huang, Shi Zhang, Wei Chang, Feiping Xia, Songqiao Liu, Yi Yang, Haibo Qiu

**Affiliations:** Department of Critical Care Medicine, Zhongda Hospital, School of Medicine, Southeast University, Nanjing, Jiangsu China

**Keywords:** Catecholamine, Terlipressin (TP), Adults, Shock, Septic

## Abstract

**Background:**

Catecholamines are the first-line vasopressors used in patients with septic shock. However, the search for novel drug candidates is still of great importance due to the development of adrenergic hyposensitivity accompanied by a decrease in catecholamine activity. Terlipressin (TP) is a synthetic vasopressin analogue used in the management of patients with septic shock. In the current study, we aimed to compare the effects of TP and catecholamine infusion in treating septic shock patients.

**Methods:**

A systematic review and meta-analysis was conducted by searching articles published in PUBMED, EMBASE, and the Cochrane Central Register of Controlled Trials between inception and July 2018. We only selected randomized controlled trials evaluating the use of TP and catecholamine in adult patients with septic shock. The primary outcome was overall mortality. The secondary outcomes were the ICU length of stay, haemodynamic changes, tissue perfusion, renal function, and adverse events.

**Results:**

A total of 9 studies with 850 participants were included in the analysis. Overall, no significant difference in mortality was observed between the TP and catecholamine groups (risk ratio(RR), 0.85 (0.70 to 1.03); *P* = 0.09). In patients < 60 years old, the mortality rate was lower in the TP group than in the catecholamine group (RR, 0.66 (0.50 to 0.86); *P* = 0.002). There was no significant difference in the ICU length of stay (mean difference, MD), − 0.28 days; 95% confidence interval (CI), − 1.25 to 0.69; *P* = 0.58). Additionally, TP improved renal function. The creatinine level was decreased in patients who received TP therapy compared to catecholamine-treated participants (standard mean difference, SMD), − 0.65; 95% CI, − 1.09 to − 0.22; *P* = 0.003). No significant difference was found regarding the total adverse events (Odds Ratio(OR), 1.48(0.51 to 4.24); *P* = 0.47), whereas peripheral ischaemia was more common in the TP group (OR, 8.65(1.48 to 50.59); *P* = 0.02).

**Conclusion:**

The use of TP was associated with reduced mortality in septic shock patients less than 60 years old. TP may also improve renal function and cause more peripheral ischaemia. PROSPERO registry: CRD42016035872.

## Background

Sepsis and septic shock are a grave consequence of infection, and the mortality is high [1, 2], despite the significant progress made in intensive care medicine. Volume resuscitation is the mainstay approach for management of septic shock, followed by vasoactive infusions to maintain appropriate arterial pressure and tissue perfusion. Early resuscitation in septic shock could raise the mean arterial pressure (MAP) to facilitate the tissue perfusion of organs and enhance the oxygen supply [3, 4].

No statistical significance has been shown in the survival benefit of one vasopressor over another. Norepinephrine is the first-recommended vasopressor according to the Surviving Sepsis Campaign [5]. The major cause of refractory hypotension in septic shock patients is insensitivity or no response to vasoactive agents [6]. Additionally, previous studies have reported significant adverse effects of high-dose catecholamines [7, 8].

Vasopressin (AVP) is an endogenously released stress hormone that is important during shock. Growing evidence has suggested that arginine vasopressin infusion is safe and effective, and it has been recommended as a first-line vasopressor for the treatment of septic shock [9, 10]. TP is an AVP analogue with a longer half-life (6 h) and duration of action (2 to 10 h) compared with vasopressin (half-life, 6 min; duration of action, 30 to 60 min). The preliminary clinical analysis revealed that TP effectively reduced the norepinephrine (NE) requirements of patients with septic shock [11, 12].

Studies comparing the use of TP and catecholamine showed conflicting results. In a meta-analysis, TP decreased the NE requirement and mortality rate in patients suffering from sepsis and septic shock [13]. A recent study demonstrated that there was no significant improvement in the 28-day mortality rate in patients treated with TP versus catecholamine. In this systematic review, we summarized the results from randomized controlled trials focusing on the comparison between TP and catecholamine treatments in septic shock using a meta-analysis. Our findings may provide important insights for future trial planning and the development of treatment guidelines.

## Methods

### Search for trials

This work was registered in the international prospective register of systematic reviews (PROSPERO registry number: CRD42016035872). We searched publications in the PUBMED, EMBASE and COCHRANE databases up to July 2018 using a sensitive search strategy combining the keywords and subject headings. Relevant articles were identified using the terms “shock, septic”, “terlipressin”, and “adults”. The reference lists of recent reviews and retrieved studies were examined. No date or language restrictions were used. We did not attempt to identify unpublished reports or contact authors for additional information.

### Eligibility criteria

The inclusion criteria were as follows: 1) type of study: randomized controlled trials; 2) population: adult patients (> 18 years old) with septic shock; 3) intervention: catecholamine or TP to raise blood pressure; and 4) outcomes: a) primary outcomes: mortality at hospital discharge and b) secondary outcomes: length of ICU stay, haemodynamic indices, and renal function including the variables of serum creatine and urine volume. Studies with patients < 18 years old or without a control group were excluded.

### Study selection

Independent screening of the titles and abstracts was carried out by two researchers. The full-text articles were assessed for eligibility following the inclusion/exclusion criteria. A third researcher was solicited in case of discrepancies.

### Data extraction and outcomes

Data from all manuscripts were collected independently by three researchers using a data-recording form. Then, the extracted information was reviewed. Discrepancies among researchers were solved by consensus. All additional information was obtained from the principal investigators of the included studies.

The primary outcome was mortality (all causes) at the longest follow-up time. The secondary outcomes were the ICU length of stay, haemodynamic changes, tissue perfusion, renal function and adverse events.

### Quality assessment

The Cochrane Risk of Bias Tool was used for the quality assessment. The following domains were evaluated: sequence generation, allocation concealment, blinding, incomplete outcome data, selective outcome reporting, and other sources of bias [14]. The risk of bis was labelled as high, unclear, or low. Any disagreements were resolved by a consensus discussion. The quality of the evidence in this systematic review was rated by the Grading of Recommendation Assessment, Development and Evaluation (GRADE) instrument [15, 16].

### Statistical analysis

A random-effects model was used for the meta-analysis. The effect of the treatment on outcome measures was analysed using random-effects models. The difference between groups was shown as the pooled OR/RR with a 95% CI. For continuous outcomes, MDs/SMDs and 95% CIs were calculated. In some studies, the median value was reported as the measure of treatment effect, accompanied by the range or interquartile range (IQR). Before analysing the data, we estimated the mean from the medians and standard deviations (SDs) from the IQRs, as previously described [17]. Heterogeneity was determined using the I^2^ statistic. I^2^ < 50% indicated insignificant heterogeneity, and a fixed-effect model was used, whereas I^2^ > 50% was considered significant heterogeneity, and a random-effects model was used. In cases where heterogeneity was identified, sensitivity analyses were performed to investigate the influence of the individual studies on the overall estimate. A subgroup analysis for the primary outcome was also performed to explore the influencing factors and to evaluate the robustness of the primary outcome. The network graphs were produced in Stata 12.0 using the networkplot package. GeMTC (version 0.14.3) and OpenBUGS (version 3.2.2) were used to evaluate the effect of six therapies (vasopressin, dopamine, norepinephrine, terlipressin, TP plus norepinephrine, TP plus norepinephrine plus dopamine) on mortality. Data analyses were performed using Review Manager (Version 5.3), and *P* < 0.05 indicated statistical significance.

### Subgroup analyses

Pre-specified subgroup analyses were performed in studies enrolling patients with proven septic shock and focusing on the comparison between TP and catecholamine infusion in patients with septic shock. Elderly patients were defined as those aged more than 60 years according to the WHO. Therefore, we further separated the studies enrolling patients with an average age of ≥60 years vs. those enrolling patients with an average age of < 60 years to determine which subpopulation may benefit more from TP treatment.

## Results

### Literature search

**Fig. 1:**
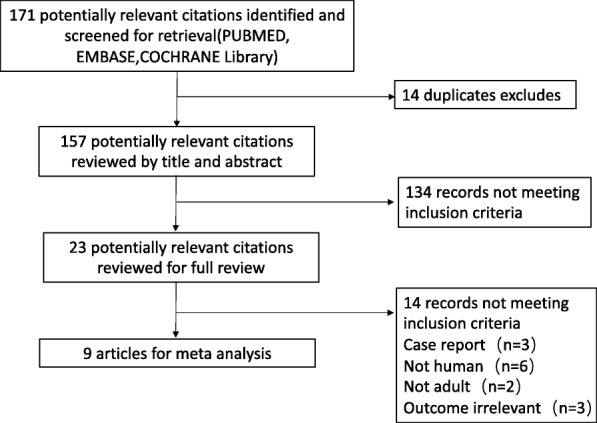
Study flow diagram

In a total of 171 citations, 148 were excluded after the initial title/abstract screening, leaving 23 articles for a full-text review. Of these, we selected 9 randomized controlled trials for the analysis (Fig. 1). Fourteen articles were excluded for the following reasons: animal studies (*n* = 6), paediatric subjects (*n* = 2), case report (*n* = 3), and outcome irrelevant (*n* = 3). Finally, 9 articles (850 patients) were included in the analysis [12, 18–25].

### Study characteristics

**Fig. 2:**
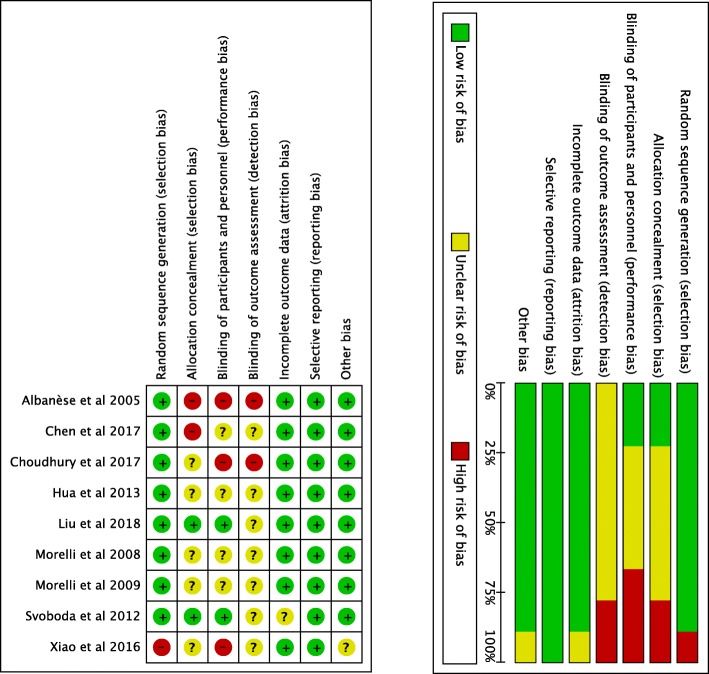
Risk of bias graph and risk of bias summary. Review authors’ judgement about each risk of bias item presented as percentages across all included studies and the authors’ judgement about each risk of bias item for each included study

The characteristics of the ten articles are presented in Table 1. Nine studies (850 patients) were included, in which 421 patients received TP and 429 patients received norepinephrine or dopamine. In all studies, conventional therapy with vasopressors/inotropes and volume resuscitation were given before the treatment with TP. In Fig. 2, the methodology of the quality assessment using the Cochrane Risk of Bias Tool is shown. There were three single- or double-blinded RCTs [20, 22, 25], and two open-label RCTs [18, 21]. The types of the other four RCTs were not mentioned in the articles [12, 19, 23, 24].

**Table 1:** Characteristics of the studies included in the systematic review

Study	Arms	n	Age(years)	Design	Dosage	Progonstic index	Time(hours)	MAP objective(mmHg)
Morelli et al 2009 [12]	NE	15	64 (59-72)	RCT	15μg/min	58 (52-68) (SAPS II)	48	
	NE+TP	15	67 (60-71)		1.3μg/Kg.h	62 (57-72) (SAPS II)	48	70±5
	NE+AVP	15	66 (60-74)		0.03μ/min	60 (49-66) (SAPS II)	48	
Albanese et al 2005 [18]	NE	10	65 (24-76)	RCT	0.3μg/kg.min	29 (24-31) (APACHE II)	6	65-75
	TP	10	66 (23-79)	OL	1mg bolus	28 (24-30) (APACHE II)	6	
Xiao et al 2016 [19]	NE	17	62±14	RCT	>0.5μg/kg.min		6	≥65
	NE+TP	15	63±11	SB	1.3μg/Kg.h			
Chen et al 2017 [20]	NE	26	55.7±16.1	RCT	>1μg/kg.min	20.8±5.7 (APACHE II)	72	65-75
	TP	31	58.5±17.8	SB	0.01-0.04u/min	23.1±5.2 (APACHE II)	72	
Choudhury et al 2017 [21]	NE	42	46.76±12.11	RCT	7.5-60μg/min		48	>65
	TP	42	48.29±12.53	OL	1.3-5.2μg/min		48	
Liu et al 2018 [22]	NE	266	61.09±16.20	RCT	4-30μg/min	19.09 ± 8.26 (APACHE II)	168	65-75
	TP	260	60.93±15.86	DB	20-160μg/h	19.08 ± 7.01 (APACHE II)	168	
Hua et al 2013 [24]	DA	16	52.2±14	RCT	20μg/Kg.min	17.6 ± 5.3 (APACHE II)	48	70±5
	TP	16	56.6±16.4		1.3μg/Kg.h	19.3 ± 9.6 (APACHE II)	48	
Morelli et al 2008 [23]	NE	20	67 (29-83)	RCT	0.9μg/Kg.min	59±10 (SAPS II)	4	70±5
	NE+TP	19	66 (28-84)		1mg bolus	60±12 (SAPS II)	4	
	NE+TP+DA	20	66 (37-82)		3-20μg/Kg.min	61±12 (SAPS II)	4	
Svoboda et al 2012 [25]	NE	17	75 (48-88)	RCT	>0.6μg/Kg.min		72	70±5
	NE+TP	13	70 (37-87)	DB	4mg/24h		72	

Data presented as mean ± standard deviation or median (interquartile range). *AVP* arginine vasopressin, *DA* dopamine, *DB* double-blind, *MAP* mean arterial pressure, *NE* norepinephrine, *OL* open-label, *P* placebo, *RCT* randomized controlled trial, *SB* single-blind, *TP* terlipressin

### Meta-analysis

#### Mortality in the hospital

**Fig. 3:**
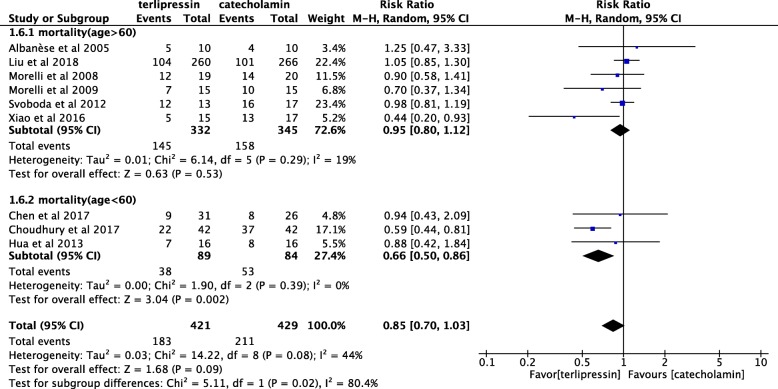
Forest plot of the effect of terlipressin compared with catecholamine on mortality in patients with septic shock as determined by a meta-analysis using a random effects model

TP infusion in patients with septic shock showed no significant impact on the mortality rate (RR, 0.85 (0.70 to 1.03); *P* = 0.09). In subgroups, the mortality rate in patients younger than 60 years old treated with TP was significantly decreased (RR, 0.66 (0.50 to 0.86); *P* = 0.002), whereas TP infusion did not influence mortality in patients older than 60 years (RR, 0.95 (0.80 to 1.12); *P* = 0.53) (Fig. 3).

**Fig. 4:**
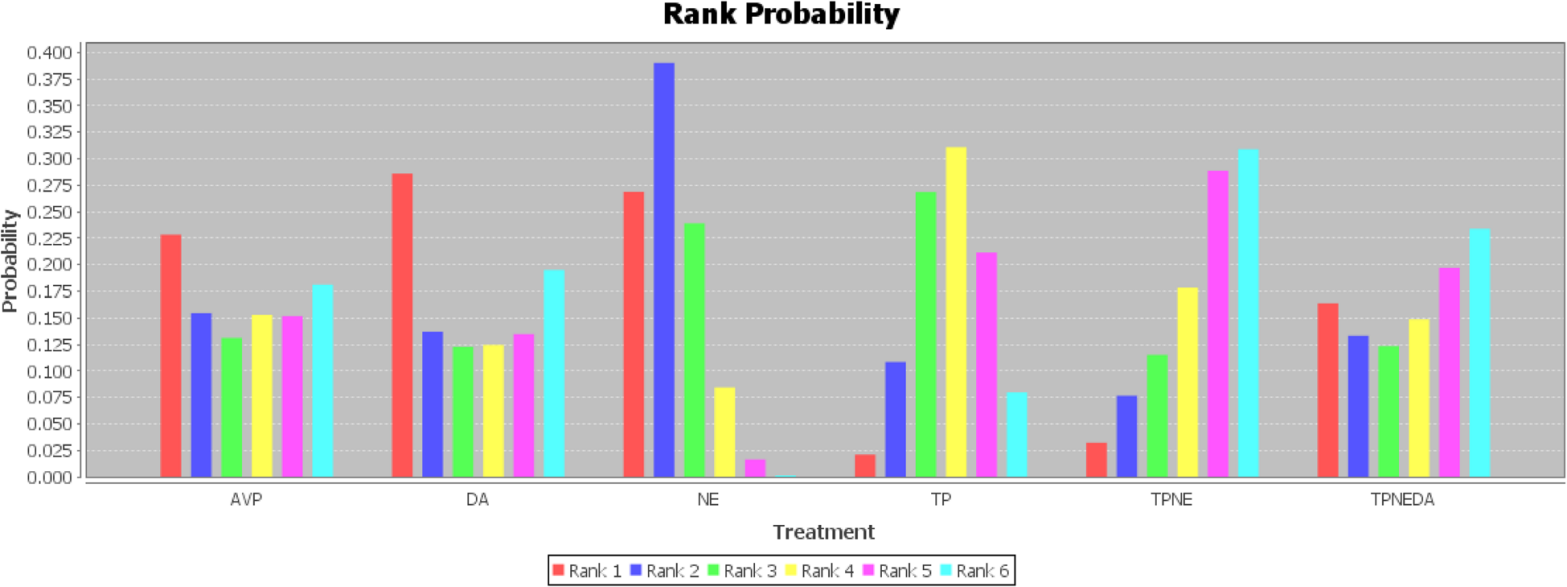
Rank probability graph of differences in mortality between different groups

The network analysis showed no significant difference in the mortality between the TP group and the other five treatment regimen groups (all CIs crossed 1) (Table S2). The ‘TP plus norepinephrine’ regimen showed the best probability of cure (31%) compared to the other regimen groups (0 to 23%) (Fig. 4).

#### Length of ICU stay

TP infusion in septic shock patients did not significantly decrease the ICU length of stay, with a pooled MD of − 0.28 days (reduction) and low heterogeneity (95% CI, − 1.25 to 0.69; I^2^ = 0%, *P* = 0.58) (Figure S1).

#### Haemodynamic variation

The addition of TP in septic shock treatment did not significantly decrease the cardiac index with a pooled SMD of − 0.19 and low heterogeneity (95% CI, − 0.58 to 0.19; I^2^ = 18%, *P* = 0.32) (Figure S2A). Compared to the catecholamine group, the TP group exhibited no significant effect on MAP variation, with a pooled SMD of 0.07 and intermediate heterogeneity (95% CI, − 0.51 to 0.66; I^2^ = 72%, *P* = 0.80) (Figure S2B). The addition of TP led to a significant reduction in the heart rate, with a pooled SMD of − 0.39 (reduction) and low heterogeneity (95% CI, − 0.73 to − 0,04; I^2^ = 44%, *P* = 0.03) (Figure S2C).

#### Tissue perfusion

The addition of TP in septic shock treatment may increase the risk of developing tissue ischaemia. Compared to the conventional treatment, TP resulted in a significant decrease in DO_2_ with a pooled SMD of − 0.58 and low heterogeneity (95% CI, − 1.15 to-0.02; I^2^ = 0%, *P* = 0.04) (Figure S3A). However, TP did not cause a significant reduction in VO_2_ (SMD, − 0.32(− 0.79 to 0.16); I^2^ = 0%, *P* = 0.20) (Figure S3B) and showed no significant effect on the Lac level (SMD, − 0.20(− 0.70 to 0.30); I^2^ = 0%, *P* = 0.43) (Figure S3C).

#### Organ function

Renal function was improved during TP infusion with the reestablishment of urine flow and a decrease in creatinine. Compared with catecholamine, TP increased the urine flow in septic shock patients with a pooled SMD of 0.49 and intermediate heterogeneity (95% CI, − 0.01 to 0.98; I^2^ = 55%, *P* = 0.05) (Figure S4A). Moreover, TP decreased the level of creatinine in patients with a pooled SMD of − 0.65 and low heterogeneity (95% CI, − 1.09 to 0.22; I^2^ = 0%, *P* = 0.003) (Figure S4B).

#### Adverse events

The pooled data displayed no significant difference in total adverse events between the two groups (OR 1.48, 95% CI, 0.51 to 4.24; I^2^ = 74%; *P* = 0.47) (Figure S5A). Arrhythmia was reported as an adverse event in three trials. However, the pooled data showed no difference in arrhythmia outcomes between the two groups (OR 0.66, 95% CI, 0.21 to 2.05; I^2^ = 32%; *P* = 0.47) (Figure S5B). Peripheral ischaemia was reported in two trials, and our pooled data showed that it was more common in the TP group (OR 8.65, 95% CI, 1.48 to 50.59; I^2^ = 71%; *P* = 0.02) (Figure S5C).

## Discussion

In this meta-analysis, we compared the use of TP and catecholamine in patients with septic shock. No significant difference was observed in the mortality risk between TP- and catecholamine-treated adult patients, which was consistent with a previous meta-analysis [26]. Furthermore, we showed, for the first time, that TP infusion was associated with a lower mortality rate in patients less than 60 years old. Previous studies found that TP caused a significantly higher rate of digital ischaemia [22], and ageing was a major risk factor for ischaemic disorders, suggesting that TP may lead to more severe digital ischaemia in elderly patients. DeBacker et al. considered microcirculatory flow as a stronger predictor of outcome [27], which might be the reason that TP did not reduce mortality in elderly patients with septic shock. Studies have also shown that TP improved oxygenation [28]. Therefore, the mortality of these patients may be lower with the use of TP.

According to the results from the network analysis and the rank probability graphs, ‘TP plus norepinephrine’ ranked first, and treatments including TP ranked in the top three. In animal models, TP treatment improved the blood flow of the kidney, intestine, and liver. Additionally, combined treatment with TP and NE was superior to TP alone [29]. Thus, the administration of TP plus norepinephrine might serve as a therapeutic option for patients with septic shock.

Haemodynamic and oxygenation variables were summarized in this meta-analysis. The TP group did not show a significant elevation in MAP or a reduction in CI and MAP. TP can reduce cardiac performance by decreasing cardiac output. In a large group of septic shock patients, TP plus norepinephrine reversed hypotension at the expense of oxygen delivery and CI [30]. In our meta-analysis, however, TP did not affect cardiac performance compared with catecholamine.

We further showed that TP significantly decreased the heart rate, indicating that TP might prevent the progression of septic shock-associated myocardial dysfunction [31]. Recent evidence suggests that diastolic dysfunction is a common symptom and a key predictor of mortality in septic shock patients [32]. Additionally, adequate ventricular filling can be achieved with a decrease in heart rate in patients with diastolic dysfunction. Unfortunately, most articles included in this review did not report the causes of septic shock or the results of echocardiography.

Our meta-analysis demonstrated that TP ameliorated renal failure, increased urine flow and decreased creatinine. TP treatment has been encouraged for hepato-renal syndrome as it significantly increased urine output compared with baseline values and promoted creatinine clearance in a prospective open-label study [30]. However, the *p*-value for the analysis of urine flow was 0.05. Our data suggested that a larger sample size of patients would be needed to reach conclusive results.

We observed that the TP and catecholamine groups had the same rate of total adverse events, which was consistent with a previously published meta-analysis [26]. Furthermore, TP was associated with a higher risk of peripheral ischaemia in comparison to catecholamine treatment. TP acts on V1 receptors, which are located on the vascular smooth muscle, leading to vasoconstriction. Thus, patients treated with TP may have a higher risk of developing tissue ischaemia [33]. However, only four of the included studies reported total adverse events, which may not completely reflect TP-related adverse events during the study period.

TP regulates vascular tone by stimulating the contraction of vascular smooth muscle cells, and TP has been used to treat hypotension in septic shock patients with catecholamine resistance [34, 35]. Emerging evidence has shown that continuous infusion of TP at low doses is effective and safe in controlling sepsis-induced arterial hypotension. TP infusion also had similar survival outcomes as other first-line vasopressor agents [12, 36–38].

There are some limitations in our meta-analysis. First, most studies had a small sample size of less than 100 participants. Small-study effects might have led to a publication bias. Second, most studies were unblended (7 of 9), which may have affected the quality of the analysis and resulted in a risk of bias. Third, significant heterogeneity was seen in some outcomes, and the dose and usage were different in these trials. Fourth, the underlying causes of septic shock varied across these studies. Finally, the earliest study was published in 2005, the latest study was published in 2018, and the definition of sepsis changed over the duration.

## Conclusions

The present meta-analysis has demonstrated the benefit of terlipressin in reducing mortality in younger patients (whose age was less than 60 years old) with septic shock. In addition, terlipressin can improve renal function in patients with septic shock, but it can also induce more peripheral ischaemia.

## Supplementary information

**Additional file 1 Figure S1.** Forest plot of the effect of terlipressin compared with catecholamine on the length of ICU stay in patients with septic shock as determined by a meta-analysis.

**Additional file 2 Figure S2.** Forest plot of the effect of terlipressin compared with catecholamine on the haemodynamic variation in patients with septic shock as determined by a meta-analysis.

**Additional file 3 Figure S3.** Forest plot of the effect of terlipressin compared with catecholamine on tissue perfusion in patients with septic shock as determined by a meta-analysis.

**Additional file 4 Figure S4.** Forest plot of the effect of terlipressin compared with catecholamine on renal function in patients with septic shock as determined by a meta-analysis.

**Additional file 5 Figure S5.** Forest plot of the adverse events of terlipressin compared with catecholamine in patients with septic shock as determined by a meta-analysis.

**Additional file 6 Table S1.** Head-to-Head Comparisons of the RRs from the Network Analysis.

## Data Availability

The datasets used and/or analysed during the current study are available from the corresponding author upon reasonable request.

## Abbreviations

APACHE: Acute Physiology and Chronic Health Evaluation
AVP: Vasopressin
CI: Confidence interval
GRADE: Grading of Recommendation Assessment, Development and Evaluation
ICU: intensive care unit
IQR: Interquartile range
MAP: Mean arterial pressure
MD: Mean difference
NE: Norepinephrine
RR: Risk ratio
SMD: Standard mean difference
SOFA: Sequential Organ Failure Assessment
TP: Terlipressin
