# Supplementary figures and images for: Terlipressin for the treatment of septic shock in adults: a systematic review and meta-analysis
Source: BMC Anesthesiol. 2020 Mar 5;20:58. doi: 10.1186/s12871-020-00965-4 (PMC7057452; doi:10.1186/s12871-020-00965-4)

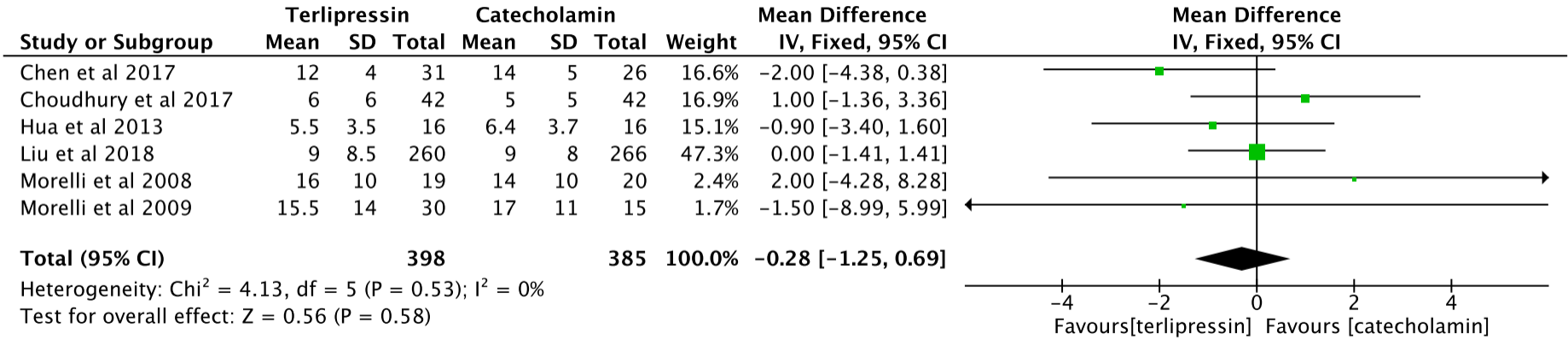

Supplement: Supplementary file 1 — Additional file 1 Figure S1. Forest plot of the effect of terlipressin compared with catecholamine on the length of ICU stay in patients with septic shock as determined by a meta-analysis. [file 12871_2020_965_MOESM1_ESM.pdf]

A

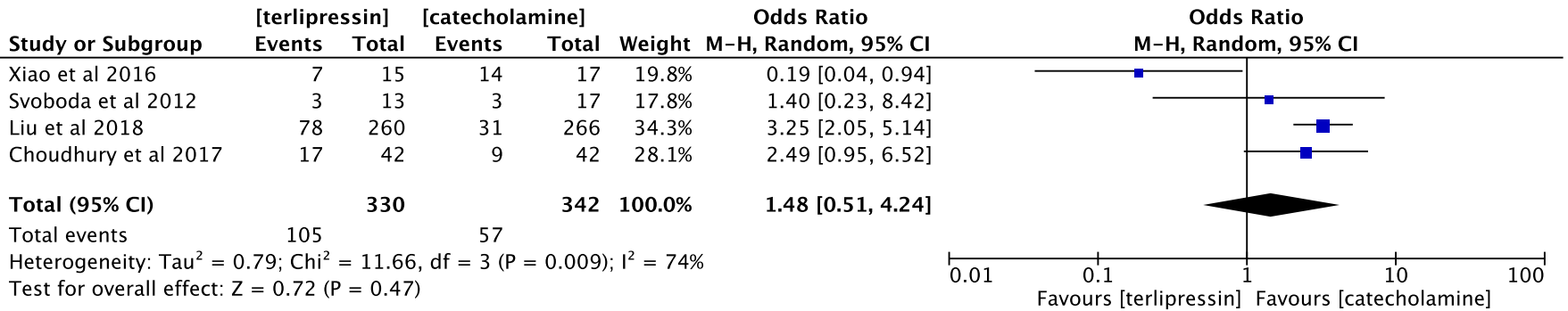

B

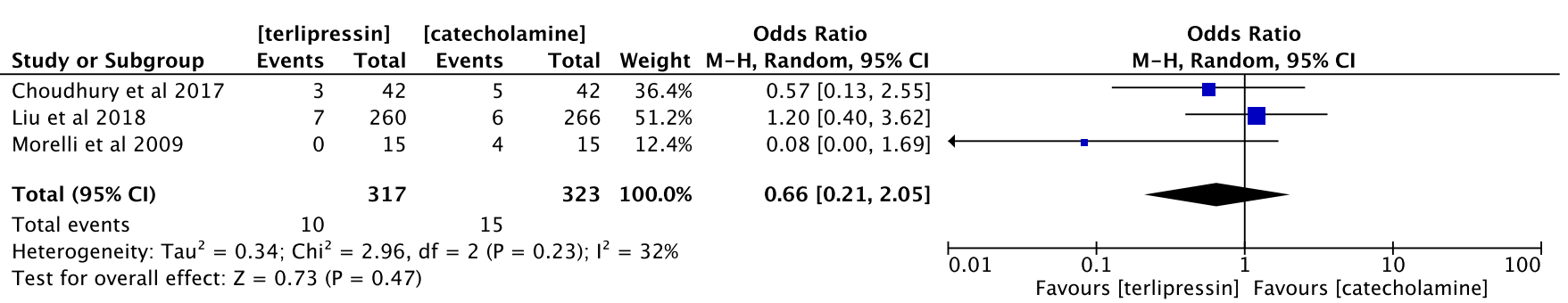

C

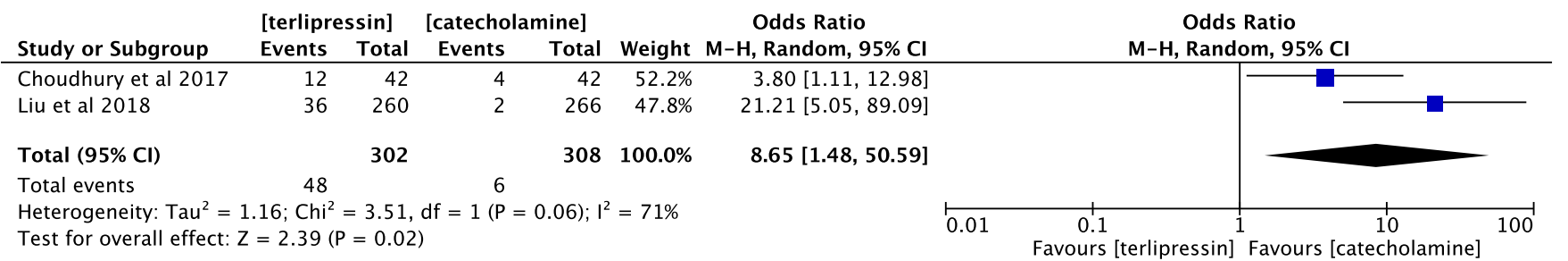

Supplement: Supplementary file 5 — Additional file 5 Figure S5. Forest plot of the adverse events of terlipressin compared with catecholamine in patients with septic shock as determined by a meta-analysis. [file 12871_2020_965_MOESM5_ESM.pdf]
