# Supplementary material for: Terlipressin for the treatment of septic shock in adults: a systematic review and meta-analysis
Source: BMC Anesthesiol. 2020 Mar 5;20:58. doi: 10.1186/s12871-020-00965-4 (PMC7057452; doi:10.1186/s12871-020-00965-4)

A

Study or Subgroup	Terlipressin Mean	Terlipressin SD	Terlipressin Total	Catecholamin Mean	Catecholamin SD	Catecholamin Total	Weight	Std. Mean Difference IV, Fixed, 95% CI
Albanèse et al 2005	−1	1.53	10	0.2	1.6	10	17.7%	−0.73 [−1.65, 0.18]
Chen et al 2017	−0.31	0.65	31	−0.35	0.62	26	54.1%	0.06 [−0.46, 0.58]
Morelli et al 2009	−0.5	0.87	15	−0.1	1.32	15	28.2%	−0.35 [−1.07, 0.37]
**Total (95% CI)**			**56**			**51**	**100.0%**	**−0.19 [−0.58, 0.19]**

Heterogeneity: $Chi^2 = 2.45$, df = 2 (P = 0.29); $I^2 = 18\%$
Test for overall effect: Z = 0.99 (P = 0.32)

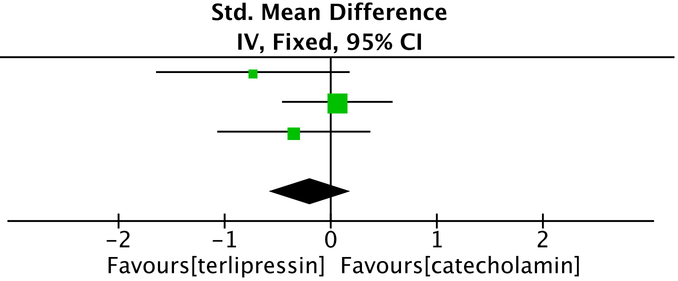

B

Study or Subgroup	[Terlipressin] Mean	[Terlipressin] SD	[Terlipressin] Total	[Catemolamin] Mean	[Catemolamin] SD	[Catemolamin] Total	Weight	Std. Mean Difference IV, Random, 95% CI
Albanèse et al 2005	15	12	10	18	13	10	17.2%	−0.23 [−1.11, 0.65]
Chen et al 2017	14	4.5	31	17.5	4.5	26	22.7%	−0.77 [−1.31, −0.23]
Morelli et al 2008	4	6	19	1	3	20	21.0%	0.62 [−0.02, 1.27]
Morelli et al 2009	18	5	15	17	3	15	19.7%	0.24 [−0.48, 0.95]
Svoboda et al 2012	1.6	8.8	13	−3.4	8.5	17	19.4%	0.56 [−0.17, 1.30]
**Total (95% CI)**			**88**			**88**	**100.0%**	**0.07 [−0.51, 0.66]**

Heterogeneity: $Tau^2 = 0.31$; $Chi^2 = 14.24$, df = 4 (P = 0.007); $I^2 = 72\%$
Test for overall effect: Z = 0.25 (P = 0.80)

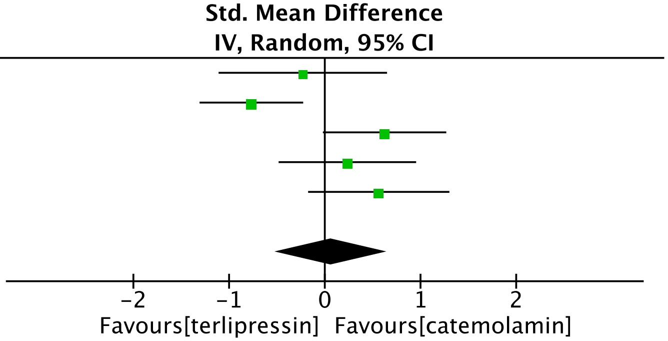

C

Study or Subgroup	Terlipressin Mean	Terlipressin SD	Terlipressin Total	Catecholamin Mean	Catecholamin SD	Catecholamin Total	Weight	Std. Mean Difference IV, Fixed, 95% CI
Albanèse et al 2005	−20	34	10	5	39	10	14.4%	−0.65 [−1.56, 0.25]
Chen et al 2017	−22.2	9.9	31	−22.9	11	26	43.5%	0.07 [−0.46, 0.59]
Morelli et al 2009	−24	16	15	−1	32	15	20.7%	−0.88 [−1.64, −0.13]
Svoboda et al 2012	−19	14	13	−6	23	17	21.4%	−0.64 [−1.39, 0.10]
**Total (95% CI)**			**69**			**68**	**100.0%**	**−0.39 [−0.73, −0.04]**

Heterogeneity: $Chi^2 = 5.37$, df = 3 (P = 0.15); $I^2 = 44\%$
Test for overall effect: Z = 2.20 (P = 0.03)

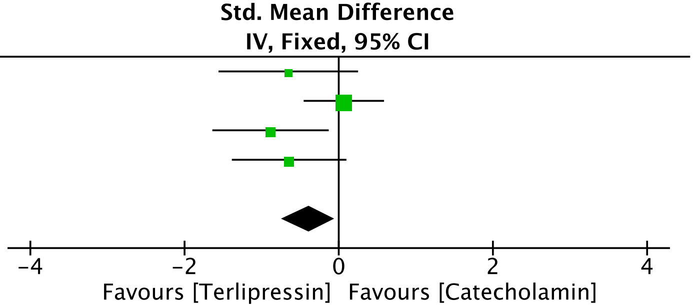

Supplement: Supplementary file 2 — Additional file 2 Figure S2. Forest plot of the effect of terlipressin compared with catecholamine on the haemodynamic variation in patients with septic shock as determined by a meta-analysis. [file 12871_2020_965_MOESM2_ESM.pdf]
