# Supplementary material for: Terlipressin for the treatment of septic shock in adults: a systematic review and meta-analysis
Source: BMC Anesthesiol. 2020 Mar 5;20:58. doi: 10.1186/s12871-020-00965-4 (PMC7057452; doi:10.1186/s12871-020-00965-4)

A

Study or Subgroup	Terlipressin Mean	Terlipressin SD	Terlipressin Total	Catecholamin Mean	Catecholamin SD	Catecholamin Total	Weight	Std. Mean Difference IV, Fixed, 95% CI
Albanèse et al 2005	−110	231	10	12	214	10	40.3%	−0.52 [−1.42, 0.37]
Morelli et al 2009	−72	91	15	7	148	15	59.7%	−0.63 [−1.36, 0.11]
**Total (95% CI)**			**25**			**25**	**100.0%**	**−0.58 [−1.15, −0.02]**

Heterogeneity: $Chi^2 = 0.03$, df = 1 (P = 0.86); $I^2 = 0\%$
Test for overall effect: Z = 2.02 (P = 0.04)

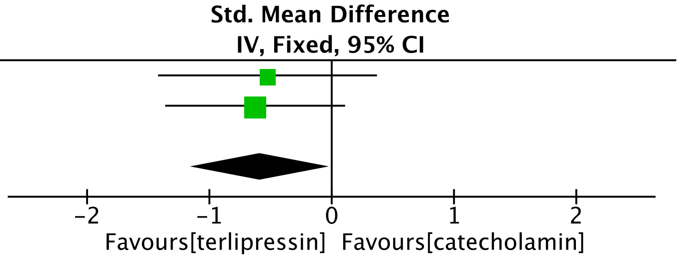

B

Study or Subgroup	Terlipressin Mean	Terlipressin SD	Terlipressin Total	Catecholamin Mean	Catecholamin SD	Catecholamin Total	Weight	Std. Mean Difference IV, Fixed, 95% CI
Morelli et al 2008	−8	46	19	−3	39	20	57.6%	−0.12 [−0.74, 0.51]
Morelli et al 2009	−32	51	15	1	58	15	42.4%	−0.59 [−1.32, 0.15]
**Total (95% CI)**			**34**			**35**	**100.0%**	**−0.32 [−0.79, 0.16]**

Heterogeneity: $Chi^2 = 0.92$, df = 1 (P = 0.34); $I^2 = 0\%$
Test for overall effect: Z = 1.30 (P = 0.20)

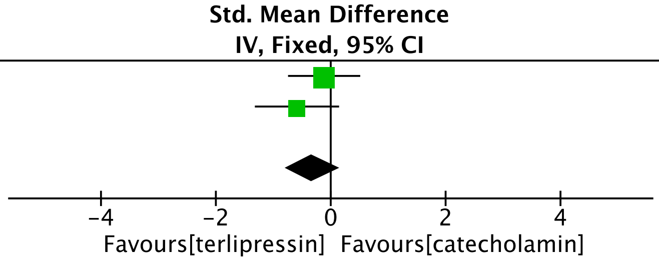

C

Study or Subgroup	[Terlipressin] Mean	[Terlipressin] SD	[Terlipressin] Total	[Catemolamin] Mean	[Catemolamin] SD	[Catemolamin] Total	Weight	Std. Mean Difference IV, Fixed, 95% CI
Morelli et al 2009	0.5	2.6	15	1.2	2.9	15	48.4%	−0.25 [−0.97, 0.47]
Xiao et al 2016	−0.5	1.7	15	−0.1	3	17	51.6%	−0.16 [−0.85, 0.54]
**Total (95% CI)**			**30**			**32**	**100.0%**	**−0.20 [−0.70, 0.30]**

Heterogeneity: $Chi^2 = 0.03$, df = 1 (P = 0.86); $I^2 = 0\%$
Test for overall effect: Z = 0.79 (P = 0.43)

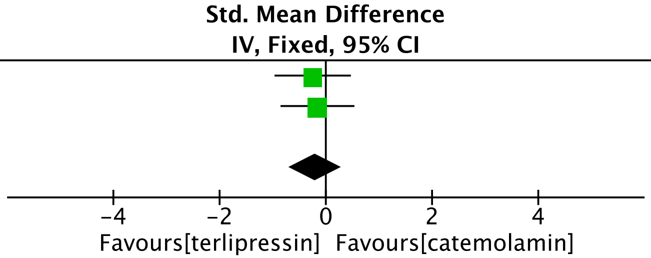

Supplement: Supplementary file 3 — Additional file 3 Figure S3. Forest plot of the effect of terlipressin compared with catecholamine on tissue perfusion in patients with septic shock as determined by a meta-analysis. [file 12871_2020_965_MOESM3_ESM.pdf]
