# Supplementary material for: Terlipressin for the treatment of septic shock in adults: a systematic review and meta-analysis
Source: BMC Anesthesiol. 2020 Mar 5;20:58. doi: 10.1186/s12871-020-00965-4 (PMC7057452; doi:10.1186/s12871-020-00965-4)

A

Study or Subgroup	Terlipressin Mean	Terlipressin SD	Terlipressin Total	Catecholamin Mean	Catecholamin SD	Catecholamin Total	Weight	Std. Mean Difference IV, Random, 95% CI
Chen et al 2017	350	693	31	103	670	26	30.1%	0.36 [−0.17, 0.88]
Morelli et al 2008	1,128	2,475	19	−168	1,117	20	25.6%	0.67 [0.02, 1.31]
Morelli et al 2009	288	776	15	480	1,327	15	23.3%	−0.17 [−0.89, 0.55]
Svoboda et al 2012	896	933	13	−143	797	17	21.1%	1.18 [0.39, 1.97]
**Total (95% CI)**			**78**			**78**	**100.0%**	**0.49 [−0.01, 0.98]**

Heterogeneity: $Tau^2 = 0.14$; $Chi^2 = 6.70$, df = 3 (P = 0.08); $I^2 = 55\%$

Test for overall effect: Z = 1.93 (P = 0.05)

Std. Mean Difference IV, Random, 95% CI

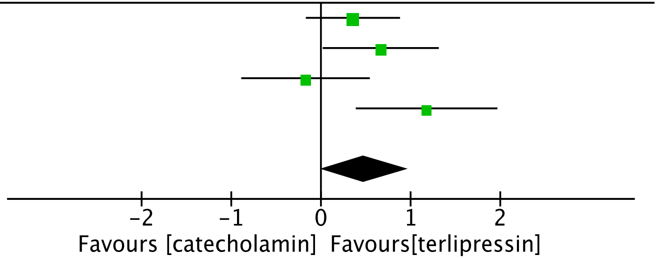

B

Study or Subgroup	Terlipressin Mean	Terlipressin SD	Terlipressin Total	Catecholamin Mean	Catecholamin SD	Catecholamin Total	Weight	Std. Mean Difference IV, Fixed, 95% CI
Chen et al 2017	−14.8	27.5	31	4.3	22.8	26	64.5%	−0.74 [−1.28, −0.20]
Morelli et al 2009	26.5	110	15	97	162	15	35.5%	−0.50 [−1.22, 0.23]
**Total (95% CI)**			**46**			**41**	**100.0%**	**-0.65 [-1.09, -0.22]**

Heterogeneity: $Chi^2 = 0.28$, df = 1 (P = 0.60); $I^2 = 0\%$

Test for overall effect: Z = 2.95 (P = 0.003)

Std. Mean Difference IV, Fixed, 95% CI

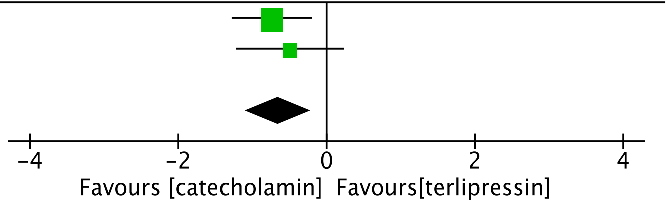

Supplement: Supplementary file 4 — Additional file 4 Figure S4. Forest plot of the effect of terlipressin compared with catecholamine on renal function in patients with septic shock as determined by a meta-analysis. [file 12871_2020_965_MOESM4_ESM.pdf]
