# Supplementary material for: Terlipressin for the treatment of septic shock in adults: a systematic review and meta-analysis
Source: BMC Anesthesiol. 2020 Mar 5;20:58. doi: 10.1186/s12871-020-00965-4 (PMC7057452; doi:10.1186/s12871-020-00965-4)
Supplement: Supplementary file 6 — Additional file 6 Table S1. Head-to-Head Comparisons of the RRs from the Network Analysis. [file 12871_2020_965_MOESM6_ESM.docx]

**Table S1. Head-to-Head Comparisons of the RRs from the Network Analysis**

| **VAP** | 1.09 (0.04,29.15) | 1.52 (0.18,14.49) | 0.86 (0.09, 7.56) | 0.58 (0.04, 8.99) | 0.78 (0.03, 18.93) |
| --- | --- | --- | --- | --- | --- |
| 0.91 (0.03, 25.90) | **DA** | 1.40 (0.10, 21.26) | 0.81 (0.06, 9.50) | 0.54 (0.03, 12.78) | 0.73 (0.02, 25.64) |
| 0.66 (0.07, 5.59) | 0.71 (0.05, 9.94) | **NE** | 0.57 (0.18, 1.52) | 0.38 (0.08, 1.78) | 0.52 (0.05, 4.72) |
| 1.16 (0.13, 10.71) | 1.23 (0.11, 15.78) | 1.76 (0.66, 5.49) | **TP** | 0.67 (0.11, 4.82) | 0.92 (0.08, 11.34) |
| 1.73 (0.11, 24.90) | 1.86 (0.08, 39.28) | 2.63 (0.56, 12.10) | 1.49 (0.21, 8.88) | **TP+NE** | 1.38 (0.15, 12.32) |
| 1.27 (0.05, 30.38) | 1.37 (0.04, 42.98) | 1.92 (0.21, 18.51) | 1.09 (0.09, 12.64) | 0.73 (0.08, 6.87) | **TP+NE+DA** |

The data on the left and below are RRs (95% CIs) of the hospital mortality in the column-defining treatment compared with the row-defining treatment. The data on the right and upper are reciprocal RRs.
